# Author response to: Comment on: Timing of symptomatic venous thromboembolism after surgery: meta-analysis

**DOI:** 10.1093/bjs/znad194

**Published:** 2023-07-05

**Authors:** Tino Singh, Jari Haukka, Quazi Ibrahim, Gordon H Guyatt, Kari A O Tikkinen

**Affiliations:** Faculty of Medicine, University of Helsinki, Helsinki, Finland; Faculty of Health Sciences, University of Eastern Finland, Kuopio, Finland; Faculty of Medicine, University of Helsinki, Helsinki, Finland; Department of Health Research Methods, Evidence and Impact, McMaster University, Hamilton, Ontario, Canada; Department of Health Research Methods, Evidence and Impact, McMaster University, Hamilton, Ontario, Canada; Department of Medicine, McMaster University, Hamilton, Ontario, Canada; Faculty of Medicine, University of Helsinki, Helsinki, Finland; Department of Urology, University of Helsinki and Helsinki University Hospital, Helsinki, Finland; Department of Surgery, South Karelian Central Hospital, Lappeenranta, Finland

*Dear Editor*

We appreciate Dr Yang for his comments on our paper, which provide us with an opportunity to clarify the methods of our study.

First, because retrospective studies often miss post-discharge venous thromboembolism (VTE) events, we included only prospective studies^1^. As surgical and perioperative practices have vastly changed over time, we included only studies with patient recruitment in the year 2000 or after. To mitigate the effect of publication bias, we included studies that had at least 20 postoperative VTE events. We included studies conducted in various surgical fields. Because we believed that timing of postoperative VTE events might not be generalizable from obstetrics, pediatric, cardiac, and neurosurgery to other surgical fields, we excluded these surgical subspecialties.

Second, in his editorial comment Dr Yang called for reporting measures of heterogeneity. Heterogenity in the outcome, incidence of VTE at each day since surgery, was explained by days since surgery (a spline), studies and their interaction using a Poisson regression. All effects were considered fixed. No natural heterogenity among studies was considered, in the Poisson regression the hypothesized rate of VTE was assumed to be constant over the study period for different studies. If authors had not reported number of events and/or population sizes, we extracted data from original papers by digitizing figures.

Finally, as studies included in our study had very consistent results, sensitivity analyses by excluding one study at a time would not have changed results.
